# Cerebral microbleeds are associated with nocturnal reverse dipping in hypertensive patients with ischemic stroke

**DOI:** 10.1186/1471-2377-14-8

**Published:** 2014-01-12

**Authors:** Hyung-Min Kwon, Jae-Sung Lim, Young Seo Kim, Jangsup Moon, Hyeri Park, Hyun Young Kim, Young-Hyo Lim, Hyunwoo Nam

**Affiliations:** 1 Seoul National University Boramae Hospital, Department of Neurology, College of Medicine, Seoul National University, Boramae-ro 5-gil 20, Dongjak-gu, Seoul 156-707, Republic of Korea; 2 Department of Neurology, College of Medicine, Hanyang University, Seoul, Republic of Korea; 3 Department of Cardiology, College of Medicine, Hanyang University, Seoul, Republic of Korea

**Keywords:** Ambulatory blood pressure monitoring, Cerebral microbleeds, Cerebrovascular disease, Cerebral ischemia, Hypertension

## Abstract

**Background:**

Abnormalities in nocturnal blood pressure dipping are well known for its relationship to cardiovascular diseases. Cerebral microbleeds are frequently observed in patients with hypertension and are known to be potent risk factors for stroke. However, there are scanty reports about the relationship between nocturnal dipping and cerebral microbleeds.

**Methods:**

We recruited consecutive patients with both hypertension and ischemic stroke within 7 days after symptom onset, and those with cardioembolism were excluded. We applied 24-hour ambulatory blood pressure monitoring two weeks after stroke onset, and we used brain MRI to detect cerebral microbleeds. Various blood pressure parameters such as mean 24-hour blood pressure, awake/sleep blood pressure, and morning surge were compared between cerebral microbleeds (+) vs. (-) groups. Subjects were further classified according to nocturnal dipping status and were analyzed by logistic regression to determine its association with cerebral microbleeds with adjustment for age, gender, and cardiovascular risk factors.

**Results:**

A total of 162 patients (100 males, age 65.33 ± 10.32 years) were included. Cerebral microbleeds were detected in 65 patients (40.1%). Most ambulatory blood pressure parameters except morning surge were significantly higher in those who had cerebral microbleeds. After adjusting for the confounding factors, the reverse dippers were prone to have cerebral microbleeds (odds ratio, 3.81; 95% confidential interval, 1.36-10.65; *p*-value = 0.01).

**Conclusion:**

Cerebral microbleeds are independently associated with reverse dipping on ambulatory blood pressure monitoring in hypertensive stroke patients.

## Background

Cardiovascular events such as stroke, myocardial infarction, and sudden cardiac death showed diurnal variations in their occurrences [1]. Based on these epidemiological data, ambulatory blood pressure monitoring (ABPM) was introduced to clinical practice and various research fields to determine the exact effect of diurnal variation of blood pressure (BP) on damage to hypertensive target organs [2-5]. Among the various reported ABPM profiles, non-dippers and reverse dippers are variably associated with cerebrovascular diseases [5-7].

Cerebral microbleeds (CMB) are known as focal accumulations of hemosiderin-containing macrophages in the perivascular spaces of small blood vessels in the brain, which result from blood extravasation, due to microvasculature structural changes in the brain caused by a high BP load [8]. This is regarded as a hypertensive target organ disease in the brain akin to leukoaraiosis and silent brain infarctions [9,10]. However, as far as we know, there are no reports evaluating the association between CMB and nocturnal dipping patterns in stroke patients. We conducted this study to evaluate the association between the nocturnal dipping pattern of blood pressure and the prevalence of CMB in the patients with both hypertension and ischemic stroke.

## Methods

### Design and Subjects

Subjects who had cerebral infarctions within one week were consecutively recruited from two university hospitals - from August 2007 to July 2011, according to the following criteria. (Table 1) Regarding exclusion criteria, we excluded stroke patients under the age of 35 because they had the possibility to have uncommon causes of stroke such as connective tissue disease, migraine-related stroke, and moyamoya disease. We also excluded patients with systolic blood pressure > 220 mmHg, or diastolic blood pressure > 120 mmHg because our practice guideline suggests antihypertensive medication in those situations and we wanted to avoid any bias by the use of antihypertensive medication during ABPM. Patients with secondary hypertension or patients who underwent intravenous thrombolysis were also excluded from the study because we could not suspend antihypertensive medications for the possible complications. Patients who suffered from severe stroke (National Institutes of Health Stroke Scale ≥ 20) were also excluded because they had the possibility of imprecise evaluation of CMB due to large-sized infarction and subsequent brain edema.

**Table 1:** Inclusion and exclusion criteria

**Inclusion criteria**	**Exclusion criteria**
1) Those from 35 to 85 years of age admitted within 7 days after stroke onset	1) Those aged < 35, or > 85
2) Those with radiologically-confirmed cerebral infarction in brain MRI (including T2*-gradient echo MRI)	2) Those with systolic blood pressure > 220 mmHg, or diastolic blood pressure > 120 mmHg
3) Those with hypertension	3) Those with secondary hypertension (eg. renal, renovascular, endocrinologic, gestational, and etc.)
4) Those who understand the study protocol and signed the informed consent	4) Night-shift workers (sleeping during the day and work from midnight to 4 AM due to job or other reasons)
	5) Those who were prescribed an intravenous recombinant tissue-plasminogen activator or anticoagulants
	6) Those who had severe stroke (NIHSS ≥ 20)
	7) Those who had life threatening medical conditions (hypertensive encephalopathy, aortic dissection, acute myocardial infarction, severe congestive heart failure and etc.)
	8) Those with confirmed cardioembolic risk factors such as atrial fibrillation

*Abbreviation:* NIHSS (National Institutes of Health Stroke Scale).

Stroke diagnosis and treatment were guided using standard guidelines, and stroke severity was assessed using the NIHSS. Each stroke etiology was defined by Trial of Org 10172 in Acute Stroke Treatment (TOAST) classifications [11]. We also included vascular risk factors such as diabetes, hyperlipidemia, coronary artery diseases, prior and current smoking history, and laboratory parameters. We defined hypertension according to the followings: history of hypertension and taking antihypertensive medications; or hypertensive target organ disease - left ventricular hypertrophy on electrocardiography or echocardiography, hypertensive retinopathy on the fundus examination; or a BP measurement of systolic BP over 140 mmHg or diastolic BP over 90 mmHg at 2 weeks post-symptom onset.

This study was approved by a local institutional review board with Boramae Hospital; additionally, we obtained written informed consents from all patients participating in the study.

### 24-hour ABPM

Due to possible reactive BP increment after stroke, we applied ABPM (Oscar 2TM Ambulatory BP monitor, SunTech Medical, NC, USA) two weeks after the ictus. Antihypertensive medications were suspended from stroke ictus per routine clinical protocol. BP was assessed every 30 minutes in the non-paretic upper extremity. Subjects who obtained less than 80% of valid BP readings were excluded.

The following parameters were calculated from raw data for the mean and standard deviations (SD): 24-hour mean systolic/diastolic BP (SBP/DBP), awake SBP/DBP, sleep SBP/DBP, morning SBP/DBP, evening SBP/DBP, lowest sleep SBP/DBP, pre-wake SBP/DBP, morning SBP surge, nocturnal BP falls, and pulse rate (awake, sleep, morning, evening, lowest, pre-wake).

Each individual parameter was defined as follows: Sleep BP was an average of all values from the beginning of sleep until awakening based on the activity sheet. Awake BP was defined as the average for the remaining time excluding sleep BP. Morning BP was designated as an average BP over 2 hours after awakening. Evening BP was an average BP during the 2 hours before bedtime. The lowest sleep BP was defined as an average BP during 1 hour that included the lowest sleep BP. Pre-wake BP was an average BP during the 2 hours before awakening.

Morning BP surge (MBPS) was defined in two different ways; First, we calculated sleep-trough MBPS as the mean SBP during the 2 hours after awakening minus the mean SBP during 1 hour including the lowest SBP during sleep [12]. Second, pre-wake MBPS was designated as the morning SBP minus the pre-wake SBP.

Nocturnal BP falls was also defined by two different means. First, the awake nocturnal fall was calculated as the awake SBP minus sleep SBP (percentile, relative awake nocturnal fall = [(1-sleep SBP/awake SBP) * 100]). Second, the evening nocturnal fall was calculated as the evening SBP minus sleep BP (percentile, relative evening nocturnal fall = [(1-sleep SBP/evening SBP) * 100]) [12].

According to the magnitude of the BP falls, subjects were divided into subgroups: dippers were those who showed ordinary nocturnal BP falls (10% ≤ nocturnal BP reductions < 20%), extreme dippers were those who had over 20% nocturnal BP falls, non-dippers were those who did not meet the ordinary magnitude of BP falls (0% ≤ nocturnal BP falls < 10%), and reverse dippers were those who showed BP increments during nighttime.

### Brain MRI

On the day of admission, diffusion-weighted imaging, apparent diffusion coefficient, axial T2, fluid-attenuated inversion recovery (FLAIR), T2*-gradient echo, sagittal T1, contrast-enhanced axial/sagittal T1, contrast-enhanced FLAIR, intracranial and extracranial MR angiography were obtained for each subject with a 1.5 Tesla superconducting magnet (MAGNETOM IMPACT EXPERT, Siemens, Germany). CMB were defined as round-shaped homogenous foci of low signal intensity lesions on T2*-weighted gradient echo images, which were less than 5 mm in diameter [13]. The foci located in the subarachnoid spaces or symmetrically in the globus pallidus were not included, because they were considered as vessel markings or calcifications. Two neurologists independently evaluated the locations and numbers of CMB, without any previous clinical information. The kappa value of agreement was 0.89, and the locations and numbers of CMB were coded based on the consensus of the two raters in the cases where there were any rater discrepancies. The locations of CMB were classified as lobar, central gray matter (basal ganglia and thalamus), and infratentorial area (brainstem and cerebellum) [14].

In addition, acute stroke locations were also reviewed on diffusion-weighted images. Lesion locations were classified as cortex, corona radiata, basal ganglia/internal capsule, and infratentorial. Furthermore, the insular cortex and amygdala were separately inspected, because there was prior evidence that disruption of those structures were associated with impaired BP variability in patients with acute stroke [15].

### Statistical analysis

We performed Chi-square tests for categorical variables, and Student’s *T*-tests for continuous variables. In order to assess the relationship between various ABPM parameters and CMB, we applied logistic regression analyses; and calculated the odds ratios at one standard deviation increment in BP parameters. Models were adjusted for age, gender, and additionally for those which showed *p*-value < 0.20 in the bivariable analysis.

## Results

A total of 162 subjects were enrolled in this study. Mean time interval between the ictus of stroke and ABPM was 15.5 days (standard deviation 4.9 days). CMB were detected in 65 (40.1%) patients. About 61% of CMB were located in the central gray matter, 27% in lobar, and 12% in infratentorial regions.

Baseline characteristics of subjects with or without CMB are described in Table 2. CMB were more common in male patients, and LDL-cholesterol was marginally lower in subjects with CMB compared to those without CMB (72.3% vs. 54.6%, *p-value* = 0.02; 107.2 ± 32.1 vs. 115.7 ± 35.1, *p-value* = 0.12; for CMB (+) vs. (-) respectively); otherwise, there were no significant differences between the two groups.

**Table 2:** Clinical and BP characteristics of study subjects

	**Total**	**CMB**		***p- *** value^*^
**Characteristics**		**Yes**	**No**	
	**(n = 162)**	**(n = 65)**	**(n = 97)**	
Age, years	65.33 ± 10.32	66.23 ± 9.47	64.73 ± 10.85	0.37
Male gender	100 (61.7)	47 (72.3)	53 (54.6)	0.02
DM	36 (22.2)	15 (23.1)	21 (21.6)	0.83
Smokers (current)	49 (30.2)	22 (34.4)	27 (27.8)	0.38
Previous statin use	15 (9.3)	6 (9.5)	9 (9.3)	0.96
Previous antiplatelet use	38 (23.5)	17 (27.4)	21 (21.9)	0.43
Previous antihypertensive medication	82 (50.6)	34 (52.3)	48 (49.5)	0.73
Total cholesterol, mg/dL	180.5 ± 41.5	174.6 ± 38.7	184.4 ± 43.1	0.14
Triglyceride, mg/dL	123.6 ± 82.0	115.0 ± 67.6	129.3 ± 90.1	0.28
LDL-C, mg/dL	112.3 ± 34.1	107.2 ± 32.1	115.7 ± 35.1	0.12
HDL-C, mg/dL	43.1 ± 11.9	44.2 ± 12.2	42.4 ± 11.7	0.35
Homocysteine, mg/dL	12.5 ± 6.2	13.2 ± 5.1	12.0 ± 6.8	0.24
Initial NIHSS	3.35 ± 3.33	3.18 ± 2.92	3.46 ±3.59	0.60
SBP, mmHg				
24-hour BP	147.1 ± 15.4	150.8 ± 16.3	144.6 ± 14.2	0.01
Awake BP	149.3 ± 15.9	152.6 ± 17.2	147.0 ± 14.6	0.03
Sleep BP	140.9 ± 17.1	145.9 ± 17.0	137.6 ± 16.5	<0.01
Morning BP	146.5 ± 17.6	149.8 ± 18.0	144.3 ± 17.2	0.05
Evening BP	149.0 ± 20.0	151.9 ± 21.5	147.0 ± 18.8	0.13
Lowest sleep BP	129.8 ± 17.8	133.1 ± 17.8	127.5 ± 17.6	0.05
Pre-wake BP	141.7 ± 18.7	146.1 ± 18.7	138.8 ± 18.2	0.01
DBP, mmHg				
24-hour BP	85.2 ± 9.3	88.8 ± 9.9	82.8 ± 8.0	< 0.01
Awake BP	86.7 ± 9.5	90.0 ± 10.2	84.4 ± 8.4	< 0.01
Sleep BP	81.3 ± 10.3	85.8 ± 10.6	78.3 ± 9.0	< 0.01
Morning BP	85.7 ± 12.1	89.7 ± 13.2	83.0 ± 10.6	< 0.01
Evening BP	86.3 ± 12.5	89.6 ± 13.1	84.1 ± 11.6	0.01
Lowest sleep BP	75.6 ± 11.0	78.6 ± 11.2	73.5 ± 10.4	<0.01
Pre-wake BP	81.3 ± 11.1	85.4 ± 11.9	79.5 ± 10.0	< 0.01

Values are presented as ‘mean ± standard deviation.’ For the categorical variables, figures denote frequencies and percentages in parentheses.

^*^Student’s *T*-tests for continuous variables and Chi-square tests for categorical variables.

Abbreviations: CMB (cerebral microbleeds), DM (diabetes mellitus), LDL-C (low density lipoprotein-cholesterol), HDL-C (high density lipoprotein-cholesterol), NIHSS (National Institutes of Health Stroke Scale), SBP (systolic blood pressure), DBP (diastolic blood pressure).

The distribution of stroke lesions is shown in Table 3. Though cortical infarctions were more common in CMB (-) patients, there were no significant differences in other locations including the insular and amygdala regions.

**Table 3:** Stroke characteristics of subjects with/without CMB

	**Total**	**CMB**		***p- *** value^*^
**Characteristics**		**Yes**	**No**	
	**(n = 162)**	**(n = 65)**	**(n = 97)**	
Stroke subtype				0.26
SVO	92 (56.8)	39 (60.0)	53 (54.6)	
LAD	49 (30.2)	17 (26.2)	32 (32.9)	
UD	20 (12.3)	9 (13.8)	11 (11.3)	
Stroke lesion location^†^				
Insular cortex	11(6.8)	4 (6.2)	7 (7.2)	0.99^‡^
Amygdala	2 (1.2)	1 (1.5)	1 (1.0)	0.99^‡^
Cortex	44 (27.2)	11 (16.9)	33 (34.0)	0.02
Corona radiata	62 (38.3)	25 (38.5)	37 (38.1)	0.97
BG/IC	50 (30.9)	19 (29.2)	31 (32.0)	0.71
Thalamus	21 (13.0)	9 (13.8)	12 (12.4)	0.78
Infratentorial	49 (30.2)	21 (32.3)	28 (28.9)	0.64

Parenthesis means proportion in percentage.

^*^Chi-square test.

^†^The sum of each cell is not 162, because one could have acute symptomatic lesions in several locations simultaneously.

^‡^Fisher’s Exact Test.

Abbreviations: CMB (cerebral microbleeds), SVO (small vessel occlusion), LAD (large artery disease), UD (undetermined).

One hundred four (64.2%) subjects had pre-existing hypertension and 58 were diagnosed with hypertension de novo. About half of the patients were prescribed antihypertensive medications before index stroke (82 (50.6%) in total; 34 (52.3%) in CMB (+) patients; 48 (49.5%) in CMB (-) patients; *p* = 0.73), and those medications were suspended in all patients after stroke.

Every individual ambulatory BP component in relation to CMB is presented in Table 2. Most of the BP parameters were significantly higher in subjects with CMB. When we classified subjects into three groups according to dipping status, there were 37 (22.8%) and 39 (24.1%) reverse dippers according to definitions (awake nocturnal falls, evening nocturnal falls; respectively) (Table 4). The risk of CMB was associated with reverse dippers with an odds ratio of 3.81 (95% confidential interval (CI), 1.36-10.65; *p* = 0.01) according to the definition of evening nocturnal BP falls, and after adjusting for age, gender, LDL, and 24-hour mean SBP/DBP. Awake nocturnal BP falls were not significantly associated with CMB. Additionally, morning surge did not show any significant differences between the two groups, according to both definitions.

**Table 4:** Ambulatory blood pressure components in relation to cerebral microbleeds

**BP component**	**CMB**		p-value^*^	**Unadjusted OR**	***p-value***^**†**^	**Adjusted OR**	***p-value***^**‡**^
	**Yes (n = 65)**	**No (n = 97)**					
Morning BP surge							
Sleep-trough	16.7 ± 13.8	16.8 ± 14.8	0.96	1.00 (0.98-1.02)	0.96	0.99 (0.97-1.02)	0.51
Pre-wake	3.7 ± 13.4	5.5 ± 14.1	0.41	0.99 (0.97-1.01)	0.41	0.99 (0.97-1.02)	0.58
Awake nocturnal BP falls			0.27^§^		0.29		0.49
Dipper	18 (27.7%)	33 (34.0%)		Ref.		Ref.	
Non-dipper	28 (43.1%)	46 (47.4%)		1.12 (0.53-2.34)	0.77	1.28 (0.57-2.90)	0.55
Reverse dipper	19 (29.2%)	18 (18.6%)		1.94 (0.82-4.59)	0.13	1.71 (0.64-4.62)	0.29
Evening nocturnal BP falls			0.04^§^		0.03		0.04
Dipper	14 (21.5%)	33 (34.0%)		Ref.		Ref.	
Non-dipper	29 (44.6%)	47 (48.5%)		1.45 (0.67-3.17)	0.35	1.62 (0.67-3.92)	0.28
Reverse dipper	22 (33.8%)	17 (17.5%)		3.05 (1.25-7.43)	0.01	3.81 (1.36-10.65)	0.01

Values are presented as mean ± standard deviation.

For the nocturnal BP falls, figures denoted absolute number of cases and percentage in parentheses.

^*^Independent *T*-test, unless otherwise specified.

^†^Logistic regression analysis. Data are presented as the odds ratios (OR) and 95% confidence interval.

^‡^Adjusted for age, gender, LDL, 24-hour mean SBP/DBP.

^§^Chi-square test.

Abbreviations: CMB (cerebral microbleeds), BP (blood pressure).

## Discussion

In this present work, we demonstrated that acute ischemic stroke patients who had a reverse-dipping phenomenon were prone to have CMB. Although most of the ABPM parameters lost their significances after adjusting for the cardiovascular risk factors and 24-hour mean SBP/DBP, the association between reverse dipping and CMB was still significant.

There are few reports that deal with the pathophysiologic link between dipping and CMB. Pathologically, CMB are located around the deep perforating arteries showing moderate to severe lipohyalinosis and amyloid deposits [8]. Ruptured arteriosclerotic vessels were also associated with CMB [7]. Based on these pathologic backgrounds, we are able to suggest that sustained hypertensive stress to vascular endothelium, due to reverse dipping during nighttime, could be a plausible explanation for the higher prevalence of CMB, though it needs to be confirmed by further pathologic studies.

In our results, the significant differences were observed only in the reverse dipping with the evening nocturnal BP falls, not the awake nocturnal BP falls that is the conventional definition of nocturnal dipping. Evening nocturnal BP falls were calculated with evening BP during 2 hours prior to bedtime [12]. We have developed this definition a priori by application of Kario’s definition for ‘nocturnal BP fall (mmHg)’. The reason why we have added novel definition was that comparing sleep BP with evening BP (2 hours right before falling asleep) rather than entire awake BP might be more reasonable to reflect the consequences of nocturnal BP changes, i.e. more abrupt rise or fall of BP in a short period of time. Although this definition is different from conventional one, non-dippers defined by ‘evening nocturnal BP falls’ also showed nocturnal BP increment without normal dipping pattern, which shared the similar biological meaning with conventional definition. Although this definition was not used widely, we cautiously suggest that this could be another way of inspection for nocturnal dipping.

The exact mechanism of reverse dipping is open to validation. Several explanations such as persistent activation of autonomic nervous system, sympathetic tone, and α1-adrenergic activities during the night may account for this [16]. Sheer stress to the atherosclerotic cerebral vessel walls increased adrenergic activity, platelet activity, and blood viscosity in the early morning could induce hypercoagulability; and these could further affect increased vulnerability to stroke [17]. Lesion locations also could be possible contributing factors for such phenomena. Patients who had insular lesions showed reverse dipping and increased norepinephrine serum levels [15]. However, in the current study, the frequencies of insular lesions were not associated with ABPM profiles.

The prevalence of CMB was higher than the previous studies in hypertensive patients [18], cross-sectional cohorts [19], or lacunar stroke population [20]. However, considering the prevalence of CMB of patients with stroke in general [21], it is comparable with the previous reports, which suggested that the reported prevalence of CMB was 34% (95% CI 31–36) in people with ischemic stroke. The prevalence of CMB in Korean hypertensive stroke patients was 64.7% in the previous hospital-based study [22].

In the previous studies, 63 (11.0%) patients among 575 subjects with sustained hypertension who aged over 50 years were reverse dippers [6]. In the patient with acute ischemic stroke, one study showed that 14 patients of 35 subjects (40.0%) were reverse dippers [23]. Another study showed that reverse dippers were 44.4% of non-lacunar stroke and 37.1% of lacunar stroke among consecutive patients with acute ischemic infarctions within 24 hours of symptom-onset [24]. One study that recorded blood pressure every 4 hours during 48 hours following acute stroke showed 36.9% (64 among 173 subjects) were reverse dippers [25]. Though our results might be comparable with or somewhat lower than the previous studies with acute ischemic stroke, the prevalence seemed to vary according to underlying cardiovascular risk factors and the timing of BP measurement.

There are several limitations in our study. A case can be made to call into question the eligibility criteria of our study. For the practical reason, we limited our study to hypertensive patients. CMB were regarded as hypertensive target organ disease [22], and more frequently observed in the hypertensive patients. We assumed that the effect size of dipping status on the CMB was modest; we limited our study to the hypertensive stroke patients to investigate the effect of dipping status on the CMB. If normotensive patients were included, the presence of hypertension might become another confounder for our analysis. Thus, generalizability issue should be considered when our results are interpreted.

After stroke, BP can show a reactive elevation due to increased catecholamine levels and previous hypertension and pain [18]; so the results could be biased because of symptomatic stroke. However, we applied ABPM to the subjects at least two weeks apart from their ictus. One study using ABPM showed that BP normalized to the control value one week after ictus of thromboembolic, lacunar infarctions, and intracerebral hemorrhages [26]. In another study, the subjects with lacunar and large artery atherosclerosis infarctions showed a spontaneous SBP drop in the first few hours [27]. We also considered the confounding effect of rebound hypertension due to medication suspension after acute stroke. There were no significant differences in previous antihypertensive medication history between CMB (+) vs. (-) groups. When we added this factor to our final adjusted model, OR was not compromised and the significance remained (OR 3.81; 95% CI, 1.36-10.65; *p* = 0.01).

Physical dependence caused by recent stroke could be another confounding factor. However, when we compared stroke severities using initial NIHSS between two groups, there were no significant differences in the stroke severity.

The small number of subjects should also be considered. Though there were statistical significances, the small numbers of patients in the subgroups of dippers and reverse dippers could actually weaken the strength of our conclusion. The possibility of increased type I error is another consideration of our study. When we readjusted minimum p-value for statistical significances using Bonferroni methods, the statistical significances were mostly compromised. Considering the conservative nature of Bonferroni correction, there were some possibilities that true associations were also compromised by this correction. However we admit the possibility of increased type I error, we thought that our results should be validated through hypothesis-driven research with larger sample size.

For practical reasons, we applied ABPM only once for each patient. Ideally, a repetitive ABPM application would increase data reliability; however, patient discomfort and high application cost were too much to bear in actual practice. In addition to that, the possible confounding factors such as sleep apnea were not measured. Finally, the cause-effect association cannot be referred in the present study.

## Conclusion

In conclusion, our study revealed that reverse dipping in ABPM was significantly associated with high prevalence of CMB in hypertensive patients with ischemic stroke. This has an implication of underscoring the importance of sustained nighttime hypertension for its association with CMB, which represents hypertensive target organ disease. Nighttime reverse dipping often goes unnoticed by patients, and, or physicians. Strategies to overcome these silent issues might be helpful in preventing CMB-associated cerebrovascular diseases. In future research, these associations should be reproduced and confirmed in more generalized subjects.

## Competing interests

None of the authors have any conflict of interest.

## Authors’ contributions

HMK and HN participated in the design of the study. HMK, JSL, YSK, JM, HP, HYK, YHL and HN collected the data. HMK and JSL performed the statistical analyses and drafted the manuscript. All authors read and approved the final manuscript.

## Pre-publication history

The pre-publication history for this paper can be accessed here:

http://www.biomedcentral.com/1471-2377/14/8/prepub
